# Clinical diagnosis and management of spinocerebellar ataxia in a resource-constrained setting: a case report from Eastern Nepal

**DOI:** 10.1097/MS9.0000000000002654

**Published:** 2024-10-16

**Authors:** Nabin Adhikari, Popular Pokhrel, Priyanka KC, Navin Kumar Sah, Bhupendra Shah

**Affiliations:** a B.P. Koirala Institute of Health Sciences, Dharan, Nepal; b Department of Internal Medicine, B.P. Koirala Institute of Health Sciences, Dharan, Nepal

**Keywords:** case report, resource-limited setting, spinocerebellar ataxia

## Abstract

**Introduction:**

Spinocerebellar ataxias (SCA) are a diverse group of neurodegenerative disorders with autosomal dominant inheritance, primarily affecting the cerebellum and its connections. Diagnosis typically involves genetic testing, but in resource-limited settings, clinical and neuroimaging assessments become critical. This case report highlights the role of nongenetic methods in diagnosing SCA and outlines management strategies in such settings.

**Case Presentation:**

A 41-year-old male presented with a 2-year history of progressive gait imbalance, headache, and abnormal speech. He had a family history of similar symptoms in two siblings, but no significant past medical history. Clinical examination revealed scanning speech, dysmetria, and a broad-based gait. Clinical and neuroimaging findings, including cerebellar atrophy, led to a diagnosis of SCA in the absence of genetic testing. Patient management with speech, occupational, and physical therapies, showed gradual improvement.

**Discussion:**

SCA, characterized by cerebellar atrophy and a range of clinical symptoms, is typically diagnosed through genetic testing, but clinical and imaging assessments are crucial when genetic resources are limited. This case illustrates that a comprehensive clinical evaluation, including neuroimaging, can support the diagnosis of SCA even without genetic testing. Strategic management emphasizing symptomatic relief and functional improvement through a multidisciplinary approach, including regular follow-up and personalized therapy, are crucial, as evidenced by the timely improvement observed in our case.

**Conclusion:**

In resource-limited settings, a comprehensive clinical and neuroimaging assessment is essential for diagnosing spinocerebellar ataxia when genetic testing is not feasible. Effective management through multidisciplinary therapies can improve patient outcomes, underscoring the need for innovative strategies to enhance diagnostic and treatment capabilities in such environments.

## Introduction

Highlights

- Spinocerebellar ataxia belong to rare hereditary ataxias with autosomal dominant inheritance and in the absence of genetic testing; clinical evaluation and neuroimaging are essential for diagnosing spinocerebellar ataxia in resource-limited settings.
- Symptomatic relief and functional improvement through therapies like speech, occupational, and physical therapy are effective in managing SCA in these settings.
- This case highlights the need for innovative approaches to enhance diagnosis and treatment of neurodegenerative disorders in resource-constrained environments.

Spinocerebellar ataxias (SCA) are a group of neurodegenerative and heterogeneous diseases affecting the cerebellum and other interconnected regions of the brain. SCA show an autosomal dominant pattern of inheritance and have a global prevalence of 3 in 100 000. Gait ataxia and incoordination, ocular problems with dysarthria are the typical presentation in SCA, while pyramidal, extrapyramidal signs, ophthalmoplegia, and cognitive impairment may be seen in specific SCAs^1^. There are over 40 distinct types of SCA and specific subtype can only be determined by genetic testing^2^. Conclusive diagnosis can be made only with genetic testing, however neuroimaging, using computed tomography (CT) and the types of symptoms seen in affected family members, the age(s) of disease onset, progression and severity of symptoms can be used to assess SCA^3^. In this case report, we present the importance of nongenetic assessment in diagnosing SCA with its management strategies in limited-resource settings. This case report has been drafted according to CARE 2013 guidelines^4^.

## Case presentation

**Figure 1:**
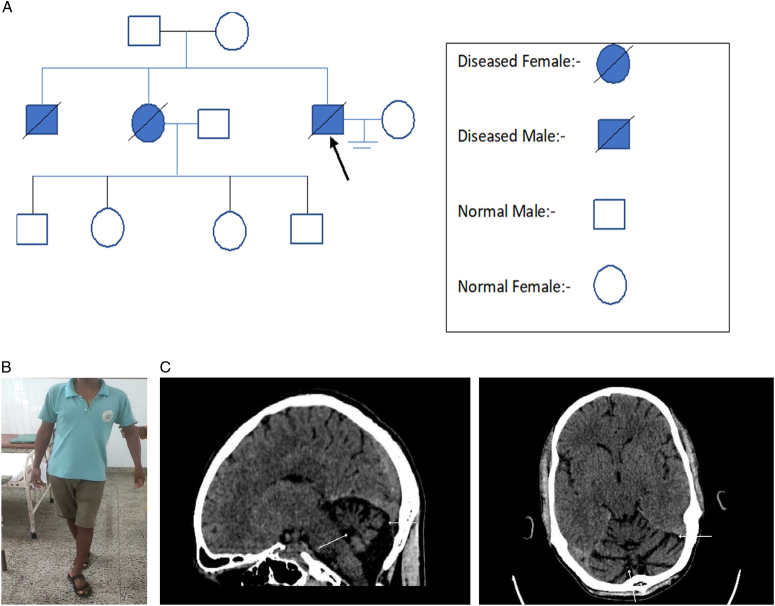
A. Pedigree analysis of the patient (patient is indicated by a black arrow) revealing an autosomal dominant pattern of inheritance, as his affected sister has all normal children. B. Demonstration of tandem walking with slight imbalance, indicative of impaired cerebellar function likely due to cerebellar atrophy. (C). The sagittal (left) and axial (right) computed tomography (CT) images revealing significant cerebellar atrophy (as indicated by the white arrow), with prominent cerebellar folia and widened surrounding CSF spaces, indicating a loss of cerebellar volume.

A 41-year-old male with no history of smoking, alcoholism, or any prior comorbidity presented to the general outpatient department of a tertiary care center with chief complaints of uncoordinated walking for the last 2 years, on and off type of headache and abnormal speech for the last 1 year. He was apparently well 2 years back when he developed an imbalance in walking, which was insidious in onset and gradually progressive of increasing severity, hampering his daily activities of living. He reported a family history of gait problems in his two siblings. His Father and Mother were normal, and one of his married sisters had all normal children, as shown in the pedigree chart [Fig. 1A]. His headache was of nonthrobbing type, mostly on the occipital region. There was no history of dizziness, light-headedness, vertigo, weight loss, fever, yellowish discoloration of sclera, exposure to environmental toxins, long-term intake of drugs, traumatic brain injury, high-risk sexual behavior, or any other significant history.

On general examination, the patient was well-built and oriented to time, person, and place. He followed all commands and had unclear speech. Pallor, icterus, cyanosis, clubbing, lymphadenopathy, edema, and dehydration were absent. His pulse rate and blood pressure measured 82 beats per minute and 120/80 mmHg, respectively. His systemic examination was unremarkable. CNS examinations revealed intact higher mental function, intact cranial nerves, motor system findings of hypotonic limbs with power 5/5 in all limbs and pupils which were bilaterally reactive to light. Mute jaw jerk, Biceps, and Triceps jerk: 1+, Supinator jerk: 2+, Knee jerk: 2+ and Pendular jerk were noted. His touch, pain, temperature, vibration sensation, and proprioception, all were intact.

Scanning type of speech with jerky and horizontal nystagmus were noted. Dysmetria by finger nose test and Dyssynergia by knee-heel test were positive. Dysdiadochokinesia was present and rebound test was positive. Broad-based gait and tandem walking were noted [Fig. 1B]. Computed Tomography (CT) scanning revealed marked cerebellar atrophy [Fig. 1C].

In our case, ataxia telangiectasia (AT) was ruled out due to the absence of several hallmark features. The patient did not exhibit the characteristic red ‘spider’ veins or other telangiectatic changes, nor did he report frequent respiratory infections, which are common in AT. Additionally, there was no history of increased sensitivity to ionizing radiation, diabetes, or premature graying of hair. Neurologically, although the patient displayed ataxia and dysmetria, he did not present with slurred speech, difficulty swallowing, or unintentional movements such as tremors, which are often seen in AT. Friedreich ataxia (FA) was also excluded based on the clinical presentation. The patient did not show any signs of cardiomyopathy or scoliosis, and sensory functions remained intact, with no loss of sensation in the arms and legs. The age of onset for the patient’s symptoms at 41 years is atypical for FA, which usually presents between the ages of five and fifteen, though it can appear later in rare cases. The absence of slurred speech beyond what was attributable to ataxia and the lack of hearing loss further supported the exclusion of FA. The diagnosis of spinocerebellar ataxia (SCA) was confirmed based on the clinical findings, the patient’s family history and neuroimaging findings. Genetic testing was not available.

A personal plan was developed to deal with symptoms of ataxia, which included speech therapy, occupational therapy, and physical therapy, and the patient has been closely worked up with regularly. The patient has had regular follow-ups since then and has been showing timely improvement.

## Discussion

Hereditary ataxia belongs to a heterogeneous group of disorders with features of uncoordinated hand and eye movements with unclear speech. Autosomal dominant and recessive are the most common patterns of inheritance^5^. Currently, hereditary ataxias are categorized into three main categories as early onset cerebellar ataxia with onset age below 25 years and autosomal recessive, autosomal dominant cerebellar ataxia with the adult onset and with either cerebellar-plus syndrome (involving additional neurological symptoms) or pure cerebellar type and idiopathic late-onset cerebellar ataxia^6^.

Most common autosomal recessive ataxias are Friedreich’s ataxia FA and ataxia telangiectasia (AT) while other very rare forms may exist. Identification of mutations in specific genes are useful in distinguishing one form from other; however clinical diagnosis can be confirmed by supplementary tests like neuroimaging and mutation analysis^7^. Most detectable symptoms in FA include gait and limb ataxia, dysarthria and loss of lower limb reflexes with deep sensory loss. Besides, cardiomyopathy, diabetes, musculoskeletal abnormalities like scoliosis, pes cavus are some of the non-neurological features seen in FA^8^. AT is a progressive neurodegenerative condition with characteristic presentations of ataxia, abnormal eye movement control, telangiectasia, and immunodeficiency. While some rare disorders may have similar presentations, selected laboratory tests and genetic sequencing is useful to reach a correct diagnosis^9^.

Spinocerebellar ataxias (SCA) have an autosomal dominant mode of inheritance with the clinical hallmarks of loss of balance and coordination and co-occurrence of speech disorder. Cerebellar atrophy is the most significant radiological finding, whereas the spinal cord, basal ganglia and other regions of the brainstem may be involved^10^. In our case, a computed tomography (CT) scan of cerebellar atrophy was seen, and this is one of the indispensable points in making the correct diagnosis.

Over 40 specific genetic SCA have been identified so far and have been classified according to the genetic loci and all variants have different presentations; thus cannot be explained at one time. Nucleotide repeat expansion, amplification, missense mutation, deletion or duplication of genes are the some of the genetic basis of etiology. Sensory and peripheral neuropathy, visual defect, macular degeneration, nystagmus, ocular muscle weakness are some of the ocular features seen in SCA. While pyramidal signs like muscle weakness, overactive reflex response, spasticity are observed, uncommon movements, psychiatric problems, and auditory loss are some of the overlapping findings seen in various types of SCA^1^.

In our case, a 41-year-old male presented with progressive imbalance, headache, and abnormal speech. The gradual onset and family history of similar symptoms in his siblings suggested a hereditary ataxia. The absence of other risk factors such as smoking, alcoholism, and exposure to toxins further supported a genetic cause. CNS examination revealed hypotonic limbs, intact cranial nerves, and distinctive signs of cerebellar involvement, such as scanning speech, nystagmus, dysmetria, and a broad-based gait. The presence of pendular reflexes and a CT scan showing marked cerebellar atrophy solidified the diagnosis of SCA. The lack of telangiectasia, cardiomyopathy, diabetes, and scoliosis ruled out other differential diagnoses like Ataxia-Telangiectasia and Friedreich Ataxia. While more focus has been given to the diagnosis of SCA and its specific subtypes by genetic testing, as many of the subtypes have overlapping clinical features, diagnosing SCA in limited-resource settings can be challenging.

SCAs, being genetically heterogeneous groups of autosomal dominant, neurodegenerative disorders; require a detailed genetic and molecular analysis for resolution of genetic heterogeneity^1^. A recent survey indicated that 36 laboratories across 20 countries conducted nearly 18 000 SCA tests in the previous year, highlighting the global emphasis on molecular genetic testing for SCAs, which is essential for differential diagnosis and adequate genetic counseling^11^. The European Molecular Genetics Quality Network (EMQN) has developed guidelines focusing on pretest requirements, appropriate methodologies, and interpretation/reporting standards to enhance the quality of SCA testing^12^. While computed tomography (CT) is a useful tool in the emergency evaluation of acute ataxia to identify potential brainstem or cerebellar hemorrhage, it often lacks the specificity needed for accurate differentiation among the various types of ataxias. Conventional MRI is the most frequently performed imaging investigation in patients with ataxia, capable of revealing patterns of atrophy such as olivopontocerebellar atrophy (OPCA), cortical cerebellar atrophy (CCA), and spinal atrophy (SA) that correlate with the underlying etiology of inherited or sporadic chronic ataxias^13^. While CT imaging is effective in ruling out acute complications such as hemorrhages or mass lesions in the cerebellum and brainstem, it presents its limitations in definitively characterizing the specific type of SCA. The imaging findings, as in our case, were consistent with patterns associated with cerebellar atrophy, but they lacked the specificity needed to differentiate among the various forms of SCA. For a more precise diagnosis, genetic testing is essential, as it can confirm the specific type of SCA based on the identified mutations. However, access to genetic testing may be constrained in limited-resource settings, as in our case, and it can impede the overall diagnostic process. Alongside, our reliance on CT imaging highlights both the value and the limitations of neuroimaging in diagnosing SCA.

SCAs do not have a definitive cure and its treatment involves symptomatic relief. Anti-epileptic drugs for seizure, botulinum toxin injections for dystonia, beta-blockers, and primidone for tremors, antidepressants for depression, and levodopa for Parkinsonism are some of the drugs used frequently^14^. Rehabilitation in patients with Spinocerebellar Ataxia (SCA) emphasizes on improving quality of life through a multidisciplinary approach, including speech, occupational, and physical therapies^15^. In our case, such a multidisciplinary approach was adopted. Regular physical therapy focused on balance and coordination exercises, such as standing on one leg, side-stepping, and fall prevention strategies, notably enhanced the patient’s stability and mobility. High-intensity motor training further improved control of motor functions, reduced ataxia symptoms, and increased independence in daily activities. Despite the lack of advanced rehabilitation tools, consistent engagement in tailored exercises resulted in meaningful improvements in balance and a reduction in fall frequency, positively impacting safety and well-being.

Additionally, the patient received occupational therapy aimed at improving daily functioning through specific exercises and task-specific training, which also promoted emotional well-being. A combined approach of intensive occupational and physical therapy resulted in further improvements in motor coordination and daily living activities. Prior referral to speech-language therapy addressed speech and swallowing difficulties, focusing on enhancing articulation and swallowing function through focused interventions. By identifying challenges like slow speech rate and difficulty with liquids, strategies such as modifying food textures and oral motor exercises were introduced, improving communication and reducing risks of aspiration and malnutrition, ultimately enhancing the patient’s quality of life. However, as SCA is progressive, ongoing support and modifications are necessary. This case highlights the significance of clinical expertise and a personalized therapy plan, particularly when genetic testing and advanced resources are unavailable, with accurate diagnosis relying on clinical features, neuroimaging, and inheritance patterns.

## Conclusion

This case highlights the significance of a comprehensive clinical evaluation and multidisciplinary management approach in the diagnosis and treatment of spinocerebellar ataxia, particularly in resource-limited settings. A high index of suspicion, integrated with thorough clinical evaluation and neuroimaging, is crucial for timely diagnosis. While genetic testing may not always be feasible, symptomatic management and supportive care can significantly improve patient outcomes in such settings. Further efforts are needed to strengthen diagnostic capabilities and management methods for patients with neurodegenerative disorders in such regions.

## Ethical approval

This case report did not intervene with the patient’s treatment plans, and hence, it did not require ethical approval.

## Consent

Written informed consent was obtained from the patient for the publication of this case report and accompanying images. A copy of the written consent is available for review by the Editor-In-Chief of this journal on request.

## Source of funding

This research did not receive any specific grants from funding agencies.

## Author contribution

P.P.: conceptualization; P.K.C., N.S., and B.S.: patient management; N.A. and P.P.: writing – original draft; N.A., P.P., P.K.C., and N.S.: writing – review and editing; B.S.: visualization and supervision. All authors have read and agreed to the final version of the manuscript.

## Conflicts of interest disclosure

The authors declare no conflict of interest.

## Research registration unique identifying number (UIN)

Case reports do not need to be registered.

## Guarantor

Popular Pokhrel.

## Data availability statement

The data that support the findings of this study are available from the corresponding author upon reasonable request.

## Provenance and peer review

Not commissioned, externally peer-reviewed.
